# Voluntary activation of biceps-to-triceps and deltoid-to-triceps transfers in quadriplegia

**DOI:** 10.1371/journal.pone.0171141

**Published:** 2017-03-02

**Authors:** Carrie L. Peterson, Michael S. Bednar, Anne M. Bryden, Michael W. Keith, Eric J. Perreault, Wendy M. Murray

**Affiliations:** 1 Edward Hines, Jr. VA Hospital, Hines, IL, United States of America; 2 Sensory Motor Performance Program, Rehabilitation Institute of Chicago, Chicago, IL, United States of America; 3 Department of Physical Medicine & Rehabilitation Northwestern University Feinberg School of Medicine, Chicago, IL, United States of America; 4 Department of Orthopaedic Surgery and Rehabilitation, Stritch School of Medicine, Loyola University Maywood, IL, United States of America; 5 The Cleveland FES Center at MetroHealth, Cleveland, OH, United States of America; 6 Department of Biomedical Engineering, Case Western Reserve University, Cleveland, OH, United States of America; 7 Department of Orthopaedics, School of Medicine, Case Western Reserve University, Cleveland, OH, United States of America; 8 Department of Biomedical Engineering, Northwestern University, Evanston, IL, United States of America; University of Memphis, UNITED STATES

## Abstract

The biceps or the posterior deltoid can be transferred to improve elbow extension function for many individuals with C5 or C6 quadriplegia. Maximum strength after elbow reconstruction is variable; the patient’s ability to voluntarily activate the transferred muscle to extend the elbow may contribute to the variability. We compared voluntary activation during maximum isometric elbow extension following biceps transfer (n = 5) and deltoid transfer (n = 6) in three functional postures. Voluntary activation was computed as the elbow extension moment generated during maximum voluntary effort divided by the moment generated with full activation, which was estimated via electrical stimulation. Voluntary activation was on average 96% after biceps transfer and not affected by posture. Individuals with deltoid transfer demonstrated deficits in voluntary activation, which differed by posture (80% in horizontal plane, 69% in overhead reach, and 70% in weight-relief), suggesting inadequate motor re-education after deltoid transfer. Overall, individuals with a biceps transfer better activated their transferred muscle than those with a deltoid transfer. This difference in neural control augmented the greater force-generating capacity of the biceps leading to increased elbow extension strength after biceps transfer (average 9.37 N-m across postures) relative to deltoid transfer (average 2.76 N-m across postures) in our study cohort.

## Introduction

Active elbow extension is lost or impaired in individuals who sustain a cervical spinal cord injury (SCI) at or above the C7 spinal level due to complete or partial paralysis of the triceps. Elbow extension function is necessary to perform self-care tasks such as eating, reaching overhead, and assisting with pressure relief. One approach to improve elbow extension function for individuals with C5 or C6 quadriplegia is tendon transfer: a surgical procedure that reassigns a donor muscle primarily innervated above the level of injury to the insertion of the paralyzed triceps. The biceps or posterior deltoid may be donor muscles to improve elbow extension function [1–4]. For some patients, surgical prerequisites (summarized previously [1, 5]) determine whether the biceps or the deltoid is transferred. For example, active brachialis and supinator muscles with sufficient strength are required for the biceps to be transferred, and adequate shoulder stability is a requirement for the posterior deltoid to be transferred. For many patients, both the biceps and posterior deltoid are candidate donor muscles for transfer. In such cases, the decision to undergo either the biceps-to-triceps or the posterior deltoid-to-triceps transfer (referred to as biceps and deltoid transfers hereafter) is influenced by the surgeon’s experience and preference [6]. Maximum elbow extension strength is an important outcome measure after tendon transfer because strength enables individuals to perform additional activities of daily living [7]. Whether the biceps or the deltoid transfer results in greater elbow extension strength post-surgery remains unclear because strength is variable across patients and studies [3, 8–15]. Understanding factors that affect elbow extension strength in arms with biceps transfer and arms with deltoid transfer would better inform donor muscle selection and rehabilitation.

The maximum force the biceps can generate is approximately twice greater than the posterior deltoid [16, 17]. Therefore, when the biceps is transferred, elbow extension is powered by a stronger muscle relative to when the posterior deltoid is transferred. Moment-generating capacity of the transferred biceps or deltoid is determined by its maximum muscle force and moment arm. Moment arms in elbow extension are presumably similar after biceps or deltoid transfer with both tendon transfers inserting on the olecranon. If moment-generating capacity were the only contributor to maximum elbow extension strength, then arms with biceps transfer would be expected to have greater strength relative to arms with deltoid transfer. Surgical outcomes described in a prospective study that randomly assigned arms to undergo either biceps or deltoid transfer and evaluated strength via manual muscle testing post-surgery were consistent with this expectation [11]. However, when elbow extension strength has been assessed objectively via measurement of the isometric moment during maximum voluntary effort, cross-sectional studies report a wide range of moments generated after deltoid transfer, which are on average greater relative to the biceps transfer. Specifically, the only study to measure moments after biceps transfer reported the average maximum moment to be 3.7 N-m [13], which is less than moments reported after deltoid transfer (combined average is 5.3 N-m) [9, 10, 12, 14, 15]. The source of the contrasting results of the single prospective study relative to the cross-sectional studies is unclear; differences in experimental design, including the study cohorts (e.g., Revol et al. assessed individuals with biceps transfers who were not candidates for deltoid transfer) and assessment procedures may be contributing factors [11, 13].

The ability to voluntarily activate the transferred muscle is an important factor that influences elbow extension strength after tendon transfer [18], in addition to the moment-generating capacity of the muscle. Deficits in voluntary activation may also contribute to observations that the deltoid transfer can result in greater strength than the biceps transfer, and maximum moments vary widely across individuals. Evidence for deficits in voluntary activation after tendon transfers, in general, exists. For example, after tendon transfer to enable lateral pinch, individuals with quadriplegia cannot fully activate the transferred brachioradialis during maximum voluntary lateral pinch [19, 20]. Voluntary activation has neither been measured after biceps nor deltoid transfer.

We expect that inadequate re-education of the nervous system to voluntarily activate the transferred muscle affects maximum elbow extension strength in both arms with biceps transfer and arms with deltoid transfer. The purpose of this study was to quantify the ability of individuals to voluntarily activate transferred muscles during maximum isometric elbow extension. Subjects with either biceps or deltoid transfers were considered. Voluntary activation was assessed using electrical stimulation to estimate the reserve capacity of the transferred muscles beyond what each subject could achieve voluntarily. We hypothesized that voluntary activation would be greater in arms with deltoid transfer relative to arms with biceps transfer because, while the deltoid is a weaker muscle, cross-sectional studies report greater isometric elbow extensor moments after deltoid transfer relative to biceps transfer. The results of this study will elucidate to what extent elbow extension strength could be improved in both transfers via rehabilitation strategies aimed to increase voluntary activation.

## Methods

### Participants

Voluntary activation was assessed in nine individuals with SCI who had undergone either biceps or deltoid transfer (Table 1). Thirteen arms (6 deltoid transfer; 7 biceps transfer) were assessed. The individuals had undergone tendon transfer prior to study enrollment and were at least one year post-surgery. Two surgeons (M.W.K and M.S.B.) performed both types of tendon transfer and post-surgery rehabilitation was the same after either transfer (Table 2). All participants were candidates for either transfer; the transfer chosen was dictated by the surgeon’s experience and preference at the time of the surgery. Pre and post-surgery manual muscle testing and range of motion assessments were conducted using techniques described by the Medical Research Council [21] and standard occupational therapy techniques [22]. Data from two individuals were excluded from the analyses because they had undergone biceps transfer to resolve biceps contracture, and thus represented a different patient demographic. The protocol was approved by the Institutional Review Board of the Edward Hines, Jr. VA Hospital. All subjects provided written informed consent that included Health Information Portability and Accountability Act consent. This study is registered on www.clinicaltrials.gov under the study title A Comparison of Two Surgical Procedures That Restore Elbow Extension and trial registry number NCT01204736.

**Table 1:** Participant Characteristics.

Arm	Transfer Muscle to Triceps	Gender	Neuro-logical Level§ Sensory	Neuro-logical Level§ Motor	Age at Injury (years)	Age at Surgery (years)	Age at Partici-pation (years)	Pre-transfer Donor Score†	Pre-transfer Triceps Score†	Post-transfer Elbow Ext. ScoreΔ	Max. Voluntary Elbow Ext. Moment (N-m)‡
1	L—Biceps	M	C5	C6	16.2	18.5	20.8	5	2	5	13.15
2	R—Biceps		C5	C6	16.2	17.5	19.4	5	2	5	12.35
3	L—Biceps	M	C4	C5	21.5	22.5	27.4	5	0	5	9.70
4	R—Biceps		C5	C5	21.5	23.5	27.4	5	0	5	9.55
5	R—Biceps	M	C4	C5	13.6	19.1	20.8	5	3	5	15.55
6	L—Deltoid	M	C5	C6	26.3	29.3	42.3	5	0	2	4.80
7	L—Deltoid	M	C4	C5	22.8	24.8	42.5	4+	2-	2-	2.00
8	L—Deltoid	F	C2	C6	28.4	30.1	36.4	4	0	2	2.70
9	R—Deltoid		C5	C6	28.4	29.9	36.4	4	0	2+	5.10
10	L—Deltoid	F	C2	C6	43.4	44.7	53.9	4	1	4	7.35
11	R—Deltoid		C6	C6	43.4	45.7	53.9	4-	1	4-	4.70
12*	R—Biceps with Contracture	M	C3	C5	34.3	40.4	41.6	4	0	0	1.70
13*	R—Biceps with Contracture	M	C4	C5	18.5	21.5	22.6	5	0	1+	3.65

*Arms were tested, but excluded from the statistical analyses due to pre-existing biceps contracture (i.e., lacking 30 degrees of passive elbow extension or more) at the time of tendon transfer.

^**§**^American Spinal Injury Association International Standards for Neurological Classification of Spinal Cord Injury assessed by a physical therapist at the Rehabilitation Institute of Chicago at the time of participation. Neurological level indicated is the most caudal level with normal function, and which all levels above are normal.

^**†**^Manual muscle test score before transfer surgery assessed by an occupational therapist at the recruitment site.

^**Δ**^Maximum manual muscle test score for the transferred muscle in elbow extension assessed by an occupational therapist at the end of the rehabilitative period.

^**‡**^Maximum voluntary elbow extension moment computed as the greatest average value held for 0.5 seconds during a single trial in any of the functional postures.

**Table 2:** Description of Surgical Procedures.

**Biceps-to-Triceps Transfer**	
**Surgical Prerequisite**	Biceps strength of 4- or greater, active brachialis and supinator muscle, a supple elbow with near complete range of motion
**Description**	An incision was made over the anterior arm passing from the medial aspect of the biceps across the antecubital fossa. Another incision was made posteriorly over the elbow, beginning just radial to the olecranon and curving to the midline at the tip of the olecranon. In the anterior wound, dissection was carried down onto the biceps. In the posterior wound, an incision was made directly over the triceps tendon at the olecranon. A drill hole was made in the olecranon. A subcutaneous tunnel was made over the ulnar aspect of the arm, wide enough to allow passage of the biceps muscle. The biceps was passed medially around the humerus. With the elbow fully extended, the ends of the biceps tendon were brought into the hole in the olecranon. Sutures were tied and reinforced.
**Post-operative Management**	The elbow was casted in full extension for 3 to 4 weeks after surgery. Thereafter, a brace was worn and adjusted each week to allow an additional 15°of flexion. Functional activities and muscle re-education were incorporated into therapy as elbow flexion increases each week. Strength training began 3 months post-surgery.
**Posterior Deltoid-to-Triceps Transfer**	
**Surgical Prerequisite**	Posterior deltoid strength of 4- or greater, active pectoralis major muscle, a supple elbow with near complete range of motion
**Description**	Incisions were made to expose the deltoid and triceps insertions. The posterior half was detached from the deltoid tuberosity and transposed posteriorly. The central third of the triceps tendon was mobilized as a proximally based flap and turned into the proximal wound and sutured to the deltoid using a non-absorbable suture. Another non-absorbable suture was attached to the olecranon through a transverse drillhole and then cross-sutured and interwoven through the tendon transfer bed to the posterior deltoid.
**Post-operative Management**	The elbow was casted in full extension for 4 weeks after surgery. Thereafter, a brace was worn and adjusted each week to allow an additional 15°of flexion. Functional activities and muscle re-education were incorporated into therapy as elbow flexion increases each week. Strength training began 3 months post-surgery.

### Experimental protocol

**Fig 1:**
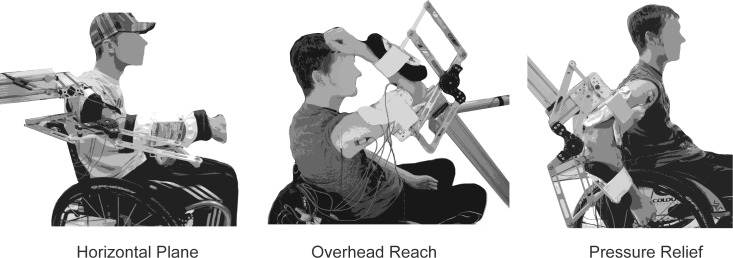
The arm was supported in each of three functional postures with an elbow moment transducer affixed to record elbow moments. The postures included the arm positioned in the horizontal plane (90° shoulder flexion, 45° shoulder internal rotation, 90° elbow flexion) and postures consistent with initiating an overhead reach (110° shoulder flexion, 70° shoulder internal rotation, 120° elbow flexion), and pressure relief (45° shoulder extension, 20° shoulder abduction, 90° elbow flexion). Shoulder and elbow joint angles followed International Society of Biomechanics recommendations [24].

Subjects completed randomized blocks of isometric elbow extension trials in each of three functional postures (Fig 1) to account for posture-dependent changes in voluntary activation and muscle moment-generating properties (force-length relationships and moment arms). Subjects were seated in their own wheelchair and positioned in each posture with their forearm in neutral and an elbow moment transducer [23] affixed to their arm. The transducer was rigidly mounted on a custom-built structure. A base supported the arm in the horizontal plane and pressure relief postures. In the overhead reach posture, the upper arm was supported by a contoured pad mounted on a lockable pivoting frame.

**Fig 2:**
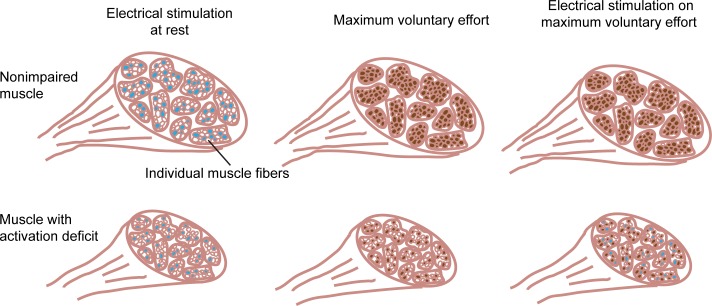
Cross-section illustration of muscle to demonstrate the recruitment of motor units during electrical stimulation, maximum voluntary effort, and electrical stimulation superimposed on maximum voluntary effort. Electrical stimulation with the muscle at rest recruits part of the motor pool (light blue fill represents muscle fibers innervated by motor units recruited by electrical stimulation). In nonimpaired muscle, nearly the entire motor pool can be voluntarily recruited at maximum effort (brown fill represents muscle fibers innervated by motor units recruited voluntarily); superposition of electrical stimulation results in additional recruitment of only a few motor units. In muscle with an activation deficit, only a percentage of the motor pool can be recruited during maximum voluntary effort; superposition of electrical stimulation recruits many additional motor units.

**Fig 3:**
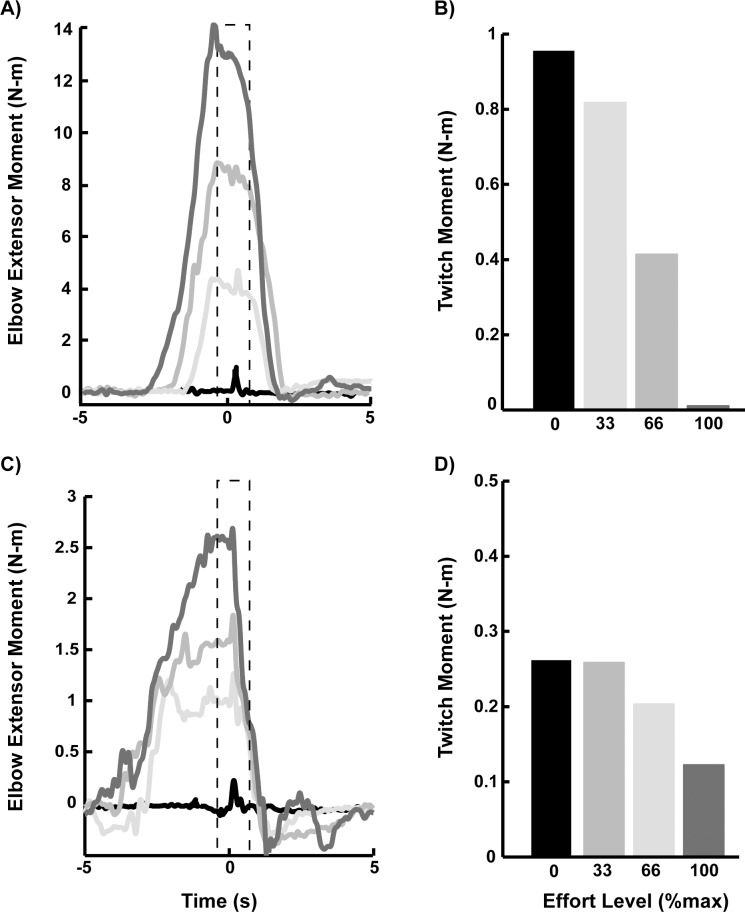
Example isometric moment-matching trials with electrical stimulation superimposed and twitch moments for two representative arms with tendon transfer. Panels (A) and (B) are Arm 2 with biceps transfer in Table 1; panels (C) and (D) are Arm 8 with deltoid transfer in Table 1). (A) and (C) Example individual trials during which electrical stimulation (applied at time 0 seconds) of the transferred muscle was superimposed on voluntary elbow extension at effort levels of 33%, 66% and 100% of maximum. For each trial, a second stimulus event was delivered at 0% of the maximum moment (black), seven seconds after the first stimulus event, but shown here at time 0 s for comparison. Twitch moments were computed as the difference in the maximum and the pre-stimulus moment within the analysis windows (dashed line). (B) and (D) Twitch moments during elbow extension and at rest corresponding to the example trials shown.

An interpolated twitch protocol [25, 26] was used to test voluntary activation of the transferred muscle during maximum isometric elbow extension. Specifically, we used electrical stimulation to estimate the percentage of the motor pool recruited during maximum voluntary effort (Fig 2). First, subjects completed three maximum voluntary elbow extension trials while moments were recorded. Maximum effort was held for 5 seconds with two minutes rest between trials. Next, two self-adhesive Ag/AgCL electrodes (Noraxon, Inc., Scottsdale, AZ) were placed on the transferred muscle (a cathode on the motor point and an anode 2.5 centimeter distal to the cathode along the muscle path) and were connected to a constant current stimulation unit (DS7AH; Digitimer Ltd., Welwyn Garden City, UK). In each posture, electrical stimulus intensity was increased in increments of 5 mA to find the minimum intensity above which further increases resulted in no change in the twitch moment (i.e., elbow extensor moment evoked by stimulation). All subsequent stimulation was delivered at 110% of the minimum intensity. Subjects were then asked to perform six moment-matching trials each at 33%, 66% and 100% of the greatest voluntary extensor moment recorded during the maximum effort trials. Subjects were provided visual feedback of their elbow moment in order to match each target moment presented in a random order. Two stimulus events (each a single pulse, 100 microseconds in duration) were delivered during each trial. The first stimulus was delivered after the subject maintained within ± 5% of the target effort level for 250 milliseconds (Fig 3). The second stimulus was delivered with the arm relaxed seven seconds after the first stimulus as a measure of the muscle response at 0% effort. Subjects were provided at least two minutes rest between maximum efforts. In six out of 33 total conditions (11 arms each in 3 postures), individuals were unable to achieve the 100% target in greater than four trials. Voltage output of the elbow moment transducer was acquired at 1000 hertz (PCI 6289; National Instruments, Austin, TX).

### Data and statistical analysis

**Fig 4:**
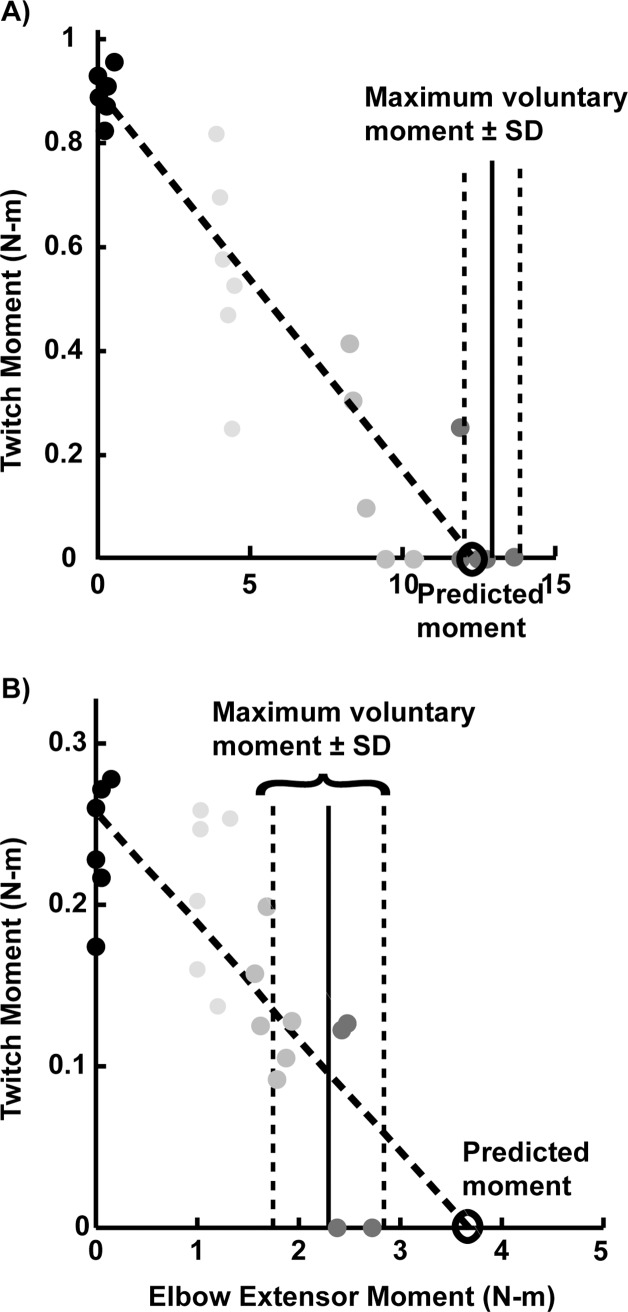
Linear extrapolation of twitch moment and voluntary pre-stimulus moment data to compute predicted moments for two representative arms with tendon transfer. (A) Arm 2 with biceps transfer in Table 1. (B) Arm 8 with deltoid transfer in Table 1.

Voluntary activation was computed as the elbow extension moment generated during maximum voluntary effort divided by the maximum moment generated with full activation, which was predicted from the experimental data (see details below). This calculation represents the ratio of the moment generated with voluntary recruitment of motor units to the moment generated with complete recruitment of the motor pool. Elbow moments were computed from the transducer’s output using a linear calibration equation (accuracy is 0.028 N-m for moments ≤ 1 N-m and 0.045% of moment for moments > 1 N-m using calibration methods described by Memberg et al.[23]). Moments were filtered using a 4^th^ order low pass digital Butterworth filter with a cutoff frequency of 80 Hz. For each maximum voluntary trial, the maximum extensor moment was computed as the greatest average moment maintained over 0.5 seconds. The pre-stimulus elbow moment and twitch moment were computed for each trial with electrical stimulation superimposed. Pre-stimulus elbow moment was computed as the average moment maintained 250 milliseconds prior to the stimulus event. The twitch moment (i.e., amplitude of the moment evoked by stimulation of muscle) was computed as the difference between the maximum elbow extensor moment occurring within 150 milliseconds after the stimulus event, and the pre-stimulus moment (Fig 3). For each arm and posture, a linear regression of twitch and voluntary moments was extrapolated to predict the moment that could be generated if the entire motor pool innervating the transferred muscle were recruited (see predicted moment in Fig 4). Because stimulation was applied at rest (i.e., 0% of maximum moment) in each trial, only the six twitch moments at rest closest to the median value were used in the regression analyses for equal representation (Fig 4). Data were excluded if the coefficient of determination of the linear fit (R^2^) was less than 0.80; three out of 33 measures of voluntary activation were excluded for this reason.

We compared mean voluntary activation between arms with biceps and deltoid transfer to test our hypothesis that voluntary activation would be greater in arms with deltoid transfer relative to arms with biceps transfer. We also tested for differences in mean voluntary moments to enable comparisons of our cohort to previous studies and evaluated the effect of posture on both voluntary activation and moments. Linear mixed-effect models and ANOVAs were used to compare mean voluntary activation and maximum extensor moments due to transfer type and posture while accounting for unequal variances and multiple comparisons (Bonferroni). Post-hoc comparisons were performed when the main effects of transfer type and posture were significant (p < .05).

## Results

**Fig 5:**
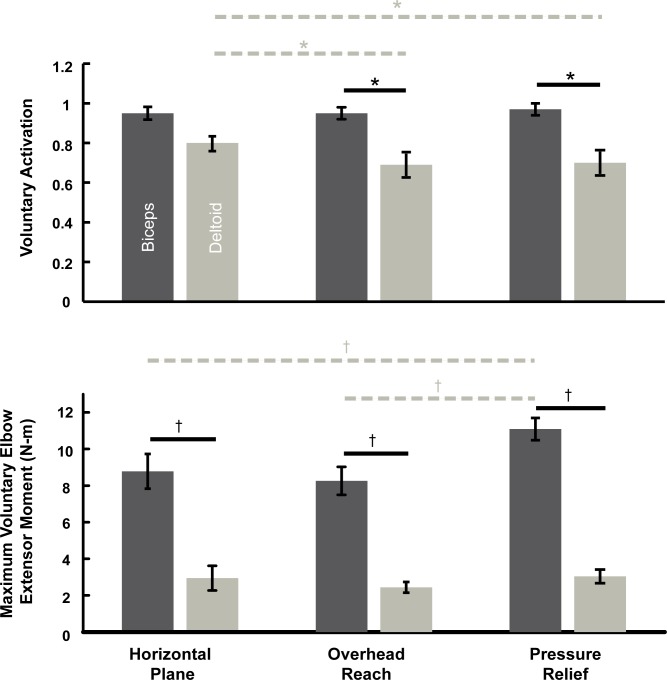
Differences in voluntary activation and maximum voluntary elbow extensor moments were detected between the biceps transfer and deltoid transfer groups. Black solid lines indicated significant differences (* indicates p < 0.05, † indicates p < 0.001). Within the deltoid transfer group, voluntary activation differed due to posture (dashed lines and * indicates p < 0.05). Within the biceps transfer group, maximum voluntary moments differed due to posture (dashed lines and † indicates p < 0.001). Error bars are ± one standard error of the means.

Maximum voluntary activation during elbow extension was greater in arms with biceps transfer relative to arms with deltoid transfer. The main effect of transfer type (F_1, 9_ = 7.1, p = .03), and the interaction of transfer type and posture (F_2, 74_ = 5.0, p = .01) were significant in the linear mixed-effect model and ANOVA of voluntary activation. Post-hoc comparisons demonstrated that voluntary activation was greater in the arms with biceps transfer relative to arms with deltoid transfer in the overhead reach (t_80_ = 3.0, p = .004) and pressure relief postures (t_80_ = 3.1, p = .002), but the difference was not significant in the horizontal plane posture (Fig 5). Voluntary activation was near complete (0.96 ± 0.02, mean ± one standard error) in the biceps transfer group, and did not change significantly across postures. In contrast, the voluntary activation of individuals with deltoid transfer exhibited a substantial dependence on posture. Within the deltoid transfer group, post-hoc comparisons demonstrated that voluntary activation was greater in the horizontal plane (0.80 ± 0.04) relative to the overhead reach (0.69 ± 0.06, t_80_ = 2.9, p = .004) and pressure relief postures (0.70 ± 0.06, t_80_ = 2.5, p = .01) (Fig 5).

Mean voluntary moments during maximum elbow extension generated by the biceps transfer arms were greater relative to voluntary moments generated by the deltoid transfer arms. The main effects of transfer type (F_1, 9_ = 28.9, p < .001) and posture (F_2, 83_ = 13.6, p < .001) were each significant in the linear mixed-effect model and ANOVA of maximum voluntary moments. Also, the interaction of transfer type and posture was significant (F_2, 83_ = 9.5, p < .001). Post-hoc comparisons demonstrated that voluntary moments generated by the biceps transfer arms were greater relative to moments generated by arms with deltoid transfer in each posture (Fig 5: all p < .001). Within the biceps transfer group, post-hoc comparisons demonstrated that the mean voluntary moments were greater in the pressure relief posture (11.09 ± 0.46 N-m, mean ± one standard error) relative to the horizontal plane (8.78 ± 0.74 N-m, t_89_ = 5.0, p < .001) and overhead reach postures (8.26 ± 0.81 N-m, t_89_ = 6.1, p < .001) (Fig 5). Within the deltoid transfer group, voluntary moments did not differ by posture. The mean elbow extensor moment generated by deltoid transfer arms across postures was 2.76 ± 0.22 N-m. The data are freely available at https://simtk.org/projects/elbowtransfer.

## Discussion

We used electrical stimulation techniques to test our hypothesis that voluntary activation during maximum isometric elbow extension would be greater in arms with deltoid transfer relative to arms with biceps transfer. Our hypothesis was not supported. Individuals with a biceps transfer were better able to activate their transferred muscle than those with a posterior deltoid transfer. This difference in neural control augmented the greater force-generating capacity of the biceps leading to increased post-surgery elbow extension strength in our study cohort. Our results demonstrate that deficits in voluntary activation can contribute to the efficacy of tendon transfer surgeries, and that these deficits influenced deltoid transfers more than biceps transfers in our study cohort.

The influence of arm posture in our study demonstrates how the interacting effects of voluntary activation and biomechanics can be confounded in measures of elbow extension strength. While arm posture did affect the ability of individuals to voluntarily activate the deltoid transfer in elbow extension, no differences in maximum elbow moments were seen across the postures we tested in the deltoid transfer arms. This result suggests the concomitant effects of these postures on moment-generating properties (e.g., force-length relationship, moment arm) for the deltoid transfer offset their effects on voluntary activation. Conversely, in the arms we tested with biceps transfer, voluntary activation was nearly complete and did not differ by arm posture. However, voluntary moments were greatest in the pressure relief posture suggesting that the moment-generating properties of the biceps transfer are advantageous in pressure relief relative to either the horizontal plane or overhead reach postures.

Greater voluntary elbow extension strength after biceps transfer relative to deltoid transfer in the arms we tested is consistent with the only previous prospective study that controlled for inter-individual and inter-arm differences by randomizing arms to undergo biceps or deltoid transfer [11]. Although elbow extensor moments were not quantified, after rehabilitation, seven out of eight arms that received biceps transfer could extend the elbow against gravity, while only one of eight arms that received deltoid transfer could extend against gravity [11]. In our study cohort, in addition to greater voluntary activation in the arms with biceps transfer and the greater force generating capacity of the biceps in general, greater triceps and pre-transfer donor muscle strength (Table 1) may have contributed to the greater strength in arms with biceps transfer relative to the arms with deltoid transfer. The biceps transfer and deltoid transfer groups in our study also differed by age, time since surgery, and gender (Table 1). Controlling for these differences was not possible due to practical reasons that limited the number of individuals in our cohort, including restrictions on recruitment, the necessity and difficulty associated with the travel of individuals with quadriplegia to our laboratory, and the reality that tendon transfer procedures are underutilized in the United States [27]. Age and time since surgery were greater in the arms with deltoid transfer we tested relative to the arms with biceps transfer (Table 1). However, based on previous long-term follow-up studies in individuals with quadriplegia and tendon transfer [28, 29], differences in age and time since surgery in our study cohort likely had a minimal effect on the difference in elbow extension strength between the arms with biceps transfer and those with deltoid transfer. Vastamaki found that elbow extension strength after deltoid transfer deteriorated by 16% in a group with an average age of 50 years who were re-evaluated 21 years post-surgery [29]. The average age of our deltoid group was 44.2 years and were 10.2 years post-surgery. Based on the study by Vastamaki, we expect that elbow extension strength deteriorated by less than 16% in our deltoid transfer group due to aging and time since surgery. However, if we conservatively estimate that the strength of the deltoid group had deteriorated by 16% between the time of surgery and participation in our study, the average elbow extension moment would have been 3.26 N-m initially after surgery and rehabilitation, which is less than the average moment we recorded in the biceps transfer arms across postures (9.37 N-m). Also, it is unlikely that age affected our results regarding voluntary activation, which does not decrease with aging in nonimpaired individuals [30]. Gender did not affect our strength measurements. The strongest two arms with deltoid transfer belonged to females.

The average maximum moment we recorded in the biceps transfer arms (8.80 N-m) in the horizontal plane exceeds the combined average of deltoid transfer arms previously reported in the horizontal plane (5.3 N-m) [9, 10, 12, 14, 15]. Thus, although the arms with deltoid transfer we tested were weaker in elbow extension relative to other cross-sectional studies that measured isometric moments [10, 12, 14], on average, we expect the biceps transfer to enable greater elbow extension strength relative to the deltoid transfer when patients are candidates for either procedure. The average maximum elbow moment we measured in the deltoid transfer arms (2.74 ± 0.22 N-m) was similar to the average of arms tested by Memberg et al. [15] (1.2 ± 2 N-m) and Kirsch et al. [9] (2.7 ± 3.5 N-m), but weaker relative to arms tested by Lieber et al. [10] (5.89 ± 0.24 N-m), Rabischong et al. [12] (6.6 ± 3.9 N-m), and Turcsanyi and Friden [14] (10.4 ± 1.0 N-m). The weaker strength we measured relative to Lieber et al. [10] and Rabischong et al. [12] may be due to differences in surgical technique and pre-transfer deltoid strength, although pre-transfer deltoid strength was not reported in these studies. Post-operative rehabilitation of the deltoid transferred arms tested by Lieber et al. [10] and Rabischong et al. [12] was the same as the rehabilitation our cohort received. Recent improvements in surgical technique that allowed for an accelerated and more intensive rehabilitation program [14] resulted in greater elbow extension strength after deltoid transfer relative to our cohort and other study cohorts. Whether more complete voluntary activation of the deltoid transfer contributed to the greater strength reported by Turcsanyi and Friden [14] remains unknown. Only one previous study has quantified maximum elbow extensor moments in arms with biceps transfer [13]. Unlike our study, the cohort examined by Revol et al. consisted of arms that were not candidates for deltoid transfer, and one arm that underwent biceps transfer after failure of a previous deltoid transfer [13]. Thus, the smaller elbow moments reported by Revol et al. [13] relative to our study is consistent with the interpretation that their cohort was more impaired by SCI than the individuals with biceps transfer we tested.

Despite our small sample size, our results regarding voluntary activation may be generalized to individuals who are candidates for either transfer that undergo the standard rehabilitation. Activation was nearly complete in all of the biceps transfer arms (ranged from 84 to 103%), whereas only two arms (one individual, Arms 10 and 11 in Table 1) with deltoid transfer achieved greater than 86% activation. Our finding that individuals with biceps transfer were better able to activate their transferred muscle can only be due to neural control. The greater voluntary activation in biceps transfer arms relative to the deltoid transfer arms suggests better re-education of the biceps to extend the elbow. Future work should determine whether better re-education of the deltoid transfer, and thereby increased voluntary activation, is achieved by improved surgical techniques and rehabilitation. Improved tendon-to-tendon attachments that allow early activation of the transferred muscle [31] may increase voluntary activation after deltoid transfer. Non-invasive rehabilitative techniques that have potential to increase voluntary activation of muscle affected by spinal cord injury include electrical stimulation of the motor cortex, spinal cord, nerve, and muscle [32–36], sensory stimulation [37], and combinations of these approaches with other neuromodulators (e.g., pharmacologic agents [34], lengthening muscle contractions [38]) and activity-based training [39].

The interpolated twitch technique has been widely used to assess voluntary activation of muscle, with a potential limitation that linear regression is used to predict the moment that could be generated with full activation. The regression predicted maximum moments that were less than voluntary moments in two conditions in arms with biceps transfer, for example as shown in Fig 4A, such that voluntary activation exceeded 100% (upper limit was 103%). However, in general, the twitch moment measurements had a good fit to the linear model, similar to many muscles studied previously (see for review [40]). Considering potential limitations of the interpolated twitch technique [40–42], electromyography (EMG) has been used to assess voluntary activation after brachioradialis transfer as a ratio of muscle activity in its post-transfer to native function [19, 20]. An EMG approach would be incorrect in arms with biceps transfer because the biceps no longer performs its native function (to supinate and flex the elbow) after transfer, such that its activation is decreased during elbow flexion relative to extension [43].

Even with complete activation, maximum elbow moments would be less in the arms with deltoid transfer relative to the arms with biceps transfer we tested. The mean voluntary moments in the biceps transfers arms were near the range of maximum elbow extensor moments individuals with preserved triceps function after SCI generate during pressure relief (11.8 to 25.6 N-m) [44] and exceeded the moments required to propel a wheelchair up a slight slope (5.7 to 7.6 N-m) [45]. In contrast, even with complete activation, the individuals with deltoid transfer we tested would be unable to generate sufficient elbow extensor moments to contribute to a pressure relief lift.

## Conclusions

Individuals with a biceps transfer were better able to activate their transferred muscle than those with a posterior deltoid transfer. Thus, motor re-education of the transferred biceps to extend the elbow was better achieved than re-education of the transferred deltoid. This difference in neural control augmented the greater force-generating capacity of the biceps relative to the posterior deltoid leading to increased post-surgery elbow extension strength in arms with biceps transfer relative to arms with deltoid transfer in our cohort.
